# Mortality related to respiratory failure among pediatric hematology patients requiring intensive care

**DOI:** 10.1186/cc12452

**Published:** 2013-03-19

**Authors:** H Okuno, K Atagi, H Shimaoka

**Affiliations:** 1 Osaka City General Hospital, Osaka, Japan

## Introduction

Although recent reports show an improvement in outcomes for pediatric hematology patients requiring intensive care [1,2], respiratory failure remains one of the major risks of pediatric mortality. This study was conducted to assess our hypothesis that mortality associated with respiratory failure is higher than that for other organ failures in pediatric hematology patients admitted to our ICU.

## Methods

A retrospective study analyzed children with hematological disorders admitted to our ICU between April 2005 and June 2012. All of the included children required emergency admission and invasive mechanical ventilation. Those who did not need intubation, or required intubation only for therapeutic intervention and died within 24 hours of ICU admission were excluded. The survival group was defined as patients who were discharged from the ICU, and the nonsurvival group was defined as those who died in the ICU or within 7 days after discharge from the ICU. The PELOD score and PIM-II were applied as morbidity scoring systems

## Results

Twenty-seven patients, including 18 males and nine females, with a median age of 6.1 years (range, 0.2 to 16.6 years) were analyzed. Sixteen patients had leukemia, five had hemophagocytic syndrome, six had solid tumors. The average predicted mortality rate was 31.3% in PIM-II. The survival group included 15 patients (56%) and the nonsurvival group included 12 patients (44%). When the survival group was compared with the nonsurvival group, there were no significant differences in the systolic blood pressure (101.3 ± 13.9 mmHg vs. 92.8 ± 25.4 mmHg; *P *= 0.15), the proportion of patients requiring continuous renal replacement therapy (33.3% vs. 50.0%; *P *= 0.30), and PELOD score (15.5 ± 10.4 vs. 21.8 ± 15.4; *P *= 0.22). In the nonsurvival group, the PIM-II was higher than that in the survival group (27.9 ± 10.4 vs. 35.7 ± 9.0; P = 0.06); the PaO_2_/FiO_2 _(272.5 ± 136.7 vs. 153.3 ± 123.3; *P *= 0.03) and oxygenation index (6.7 ± 8.1 vs. 14.1 ± 9.5; *P *= 0.04) were significantly worse in the nonsurvival group than in the survival group.

## Conclusion

The data show that respiratory failure is more strongly associated with mortality than other organ failures in pediatric hematology patients requiring intensive care. These results also suggest that mechanical ventilation intervention in patients with respiratory failure must occur earlier to improve the outcomes for these patients.
